# Focusing on optic tectum circuitry through the lens of genetics

**DOI:** 10.1186/1741-7007-8-126

**Published:** 2010-09-28

**Authors:** Linda M Nevin, Estuardo Robles, Herwig Baier, Ethan K Scott

**Affiliations:** 1 University of California, San Francisco, Department of Physiology, 1550 4th Street, San Francisco, CA 94158-23241, USA; 2 The University of Queensland, School of Biomedical Sciences, St Lucia, QLD 4072, Australia

## Abstract

The visual pathway is tasked with processing incoming signals from the retina and converting this information into adaptive behavior. Recent studies of the larval zebrafish tectum have begun to clarify how the 'micro-circuitry' of this highly organized midbrain structure filters visual input, which arrives in the superficial layers and directs motor output through efferent projections from its deep layers. The new emphasis has been on the specific function of neuronal cell types, which can now be reproducibly labeled, imaged and manipulated using genetic and optical techniques. Here, we discuss recent advances and emerging experimental approaches for studying tectal circuits as models for visual processing and sensorimotor transformation by the vertebrate brain.

## The basic 'macro-circuit' of the vertebrate visual system

**Figure 1:**
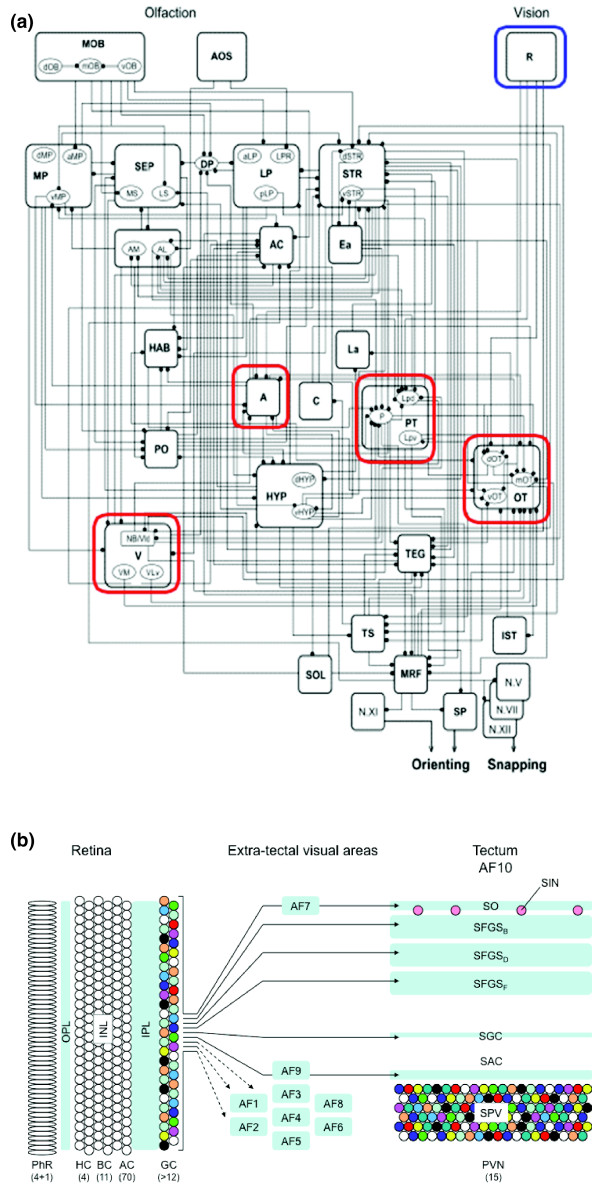
**Classical and neoclassical methods of parsing the visual system**. **(a) **Neural network underlying prey capture in anuran amphibians [3]. Anatomical studies from 1969 to 1999 were compiled to show the complex interconnectivity of visual and olfactory inputs, forebrain and midbrain contributions, and motor outputs. The retina is boxed in blue, and retinorecipient regions are boxed in red. Such schemes provide a framework for further study but do not address the pathways' micro-circuitry. A, anterior thalamus; PT, pretectum; OT, optic tectum; R, retina; V, ventral thalamus. Modified from [3]. **(b) **Scheme showing the major retinofugal connections in the larval zebrafish. Colored circles are stand-ins for diverse cell types, already known or yet to be discovered. The quantities in parentheses are estimates of the number of cell types (data compiled from work on zebrafish and other cyprinids). The retina comprises three cellular layers with five types of photoreceptors (4 cones, 1 rod), at least 11 bipolar cell types, about 70 amacrine cell types [100], and so on. The number of tectal neuron types is also large. Distinct RGC types (colors) likely have specific roles and connections with ten retinorecipient arborization fields (AF1 to AF9 plus AF10, which is the tectum) in the brain. Some anatomical details (as far as known): the RGCs that are connected to AF7 project a collateral to SO; RGC axons projecting to SAC/SPV in the tectum are routed through AF9. Abbreviations: AC, amacrine cell; AF, arborization field; BC, bipolar cell; GC, ganglion cell; HC, horizontal cell; INL, inner nuclear layer; IPL, inner plexiform layer; OPL, outer plexiform layer; PhR, photoreceptor; PVN, periventricular neuron; SAC, stratum album centrale; SFGS, stratum fibrosum et griseum superficiale; SGC, stratum griseum centrale; SIN, superficial interneuron; SO, stratum opticum; SPV, stratum periventriculare.

The transformation of visual sensory inputs into motor and endocrine responses requires specialized neural processing, often distributed across multiple structures or pathways in the brain. A classical and still vigorous branch of neuroscience, best referred to as 'functional neuroanatomy', assigns functions to specific areas in the brain. The interconnectivity of multiple areas involved in a particular sensory or behavioral task are often represented using a set of boxes, connected by arrows. The most famous such wiring diagram identified roughly 40 visual processing areas in primates [1]. Similar 'macro-circuits' have been drawn up for the visual pathway of 'lower' vertebrates [2]. In toads, a detailed circuit underlying prey capture behavior has been derived from heroic work over three decades involving tract tracing and electrophysiological mapping [3] (Figure 1a). However, none of these studies has generated a comprehensive list of essential circuit components (cell types and their connections) for a specific behavior or the processing of a specific visual stimulus. This gap in our knowledge of 'micro-circuitry' is a major obstacle to understanding the mechanisms of perception and behavior.

The zebrafish has emerged as a valuable model system with which we can hope to close this gap [4-7]. Ten different anatomical areas have been identified that serve as targets for the retinal ganglion cell (RGC) axons that connect the eye to the brain [8] (Figure 1b). These ten arborization fields, referred to as AF1 to AF10, probably correspond to the primary visual nuclei identified in adult teleost fish and are homologous to areas in mammals, such as the suprachiasmatic nuclei (AF1), the pretectal nucleus of the optic tract (AF9) and the superior colliculus/optic tectum (AF10). Not very much is known about the behavioral functions of these arborization fields in zebrafish or other fish species (with the exception of the optic tectum - see below), but it is clear that specific visual functions are initiated by activation of a fixed complement of one or very few of these nuclei [9]. Table 1 contains a comprehensive list of visually evoked behaviors reported for zebrafish.

**Table 1:** Ethogram' of zebrafish related to vision

Behavior	Description	Tectum involved?	Selected references
Visual startle	Sudden fast start following sudden changes in ambient light levels	Unknown	[62-64]
Photomotor response	Muscle contractions in response to very bright light	No	[65]
Visual background adaptation	Neuro-endocrine response of melanophore pigment cells to ambient light levels; melanin granules aggregate in bright light	No; probably AF1	[66,67]
Circadian photoentrainment	Responses in physiology and behavior to the natural light-dark cycle	No; probably AF1	[68,69]
Phototaxis	Swimming and turning toward a light source	Yes	[70-72]
Scototaxis	Preference for a dark compartment	Unknown	[73]
Dorsal light response	Tilting of the body axis toward a light source	No	[74]
Optokinetic response	Slow eye movements following the motion of a large stimulus; punctuated by saccades	No; possibly AF9	[62,67,70,75,76]; F Kubo and HB, unpublished work
Optomotor response	Turning and swimming in the direction of a large moving stimulus	No	[67,77-79]
Visually mediated dispersal	Keeping a minimum distance to other fish larvae	Unknown	AB Arrenberg and HB, unpublished work
Visual obstacle avoidance	Fast start to prevent collision with approaching object	Yes	[80]
Visual escape response	Escape turn away from any large moving object	Yes	[81]
Prey capture	Complex behavior involving J turns, slow tracking swims and fast capture swims in pursuit of small prey	Yes	[16,82-84]
Predator avoidance	Complex escape behavior; probably requires predator recognition	Yes	[85,86]
Shoaling	Grouping with conspecifics; shown by juvenile and adult fish	Unknown	[87-91]
Visual mate choice	Preference of particular shapes as reproductive stimuli by adult fish	Unknown	[92]

Here, we focus on the larval zebrafish tectum (AF10), a structure suitable for circuit analyses. The tectum sits at the surface of the brain (its name means 'roof' in Latin) and is therefore accessible to electrophysiology, laser ablations, optical imaging, and control of neuronal activity with optogenetic effectors. The tectum's broad function is known; it is involved in tasks that require a map of visual space, such as phototaxis, the approach of prey or the avoidance of obstacles (Table 1, third column). The tectum converts a visuotopic sensory map into a map of directed motor outputs. An intact tectum is dispensable for measurements of ambient light levels or for reflexes to broad moving stimuli, such as optomotor or optokinetic responses, visual background adaptation, the dorsal light reflex or photo-entrainment of circadian rhythms. In the laboratory, these behaviors can serve as negative controls for the specificity of tectum manipulations. The tectum's cellular architecture is beginning to be understood, providing an opportunity to match the structure of its micro-circuitry to its function. The zebrafish tectum is amenable to genetic manipulations. Some of the mutants and transgenic lines useful for analysis of tectal visuomotor function are summarized in Tables 2 and 3.

**Table 2:** Zebrafish mutants used for the analysis of visuomotor function

Mutant	Alleles	Phenotype	Gene	Gene product	References
*lakritz*	*lak^th241c^*	Absence of RGCs and complete blindness; no known developmental defect outside the retina	*atoh7 *(*ath5*)	Atonal homolog 7	[66]
*blumenkohl*	*blu^tc257z^*, *blu^s391^*	Synaptic transmission defect in retinotectal axons; enlarged tectal receptive fields; reduced visual acuity	*slc17a6b *(*vglut2a*)	Vesicular glutamate transporter 2a	[16]
*belladonna*	*bel^tv42^, bel^s385^*, *bel^b700^*	Incomplete crossing of retinal axons, reversed eye movements, 'looping' swim behavior	*lhx2b*	LIM-domain homeobox factor 2b	[67,93,94]
*double indemnity*	*didy^s390^*, *didy^s552^*	Reversible depletion of saccadic eye movements	*scn1lab*	Voltage-gated sodium channel NaV1.6	[60]

**Table 3:** Transgenic lines used for the analysis of tectum structure or function in zebrafish

Short name	Full name	Description	References
*Pou4f3:mGFP *(*Brn3c:mGFP*), *Pou4f3:Gal4 *(*Brn3c:Gal4*)	*Tg(pou4f3:gap43-gfp)^s356t^*, *Tg(pou4f3:gap43-gfp)^s273t^*, *Tg(pou4f3:gal4-vp16)^s311t^*	Labels a subset (40%) of RGCs; projection into SO, SFGS_D _and SFGS_F_	[10,12,22,95]
*BGUG*	*Tg(pou4f3:gal4-vp16, UAS:gap43-gfp)^s314t^*, *Tg(pou4f3:gal4-vp16, UAS:gap43-gfp)^s318t^*	Labels a random subset of Pou4f3-positive RGCs with membrane-bound GFP; also drives GFP expression in random cells within any Gal4 pattern ('genetic Golgi')	[12,20,21]
*Ath5:GFP* (*Atoh7:GFP*), *Ath5:mGFP*, *Ath5:mRFP*, *Ath5:GCaMP1.6*, *Ath5:Gal4*	*Tg(atoh7:gfp)*, *Tg(Atoh7:gap43-GFP)^cu1^, Tg(Atoh7:gap43-RFP)^cu2^*, Tg*(atoh7:gcamp1.6)*, *Tg(atoh7:gal4-vp16)*	Labels 100% of RGCs and some retinal interneurons	[42,96-98]
*Isl2b:GFP, Isl2b:mCherry-CAAX*, *Isl2b:mGFP*	*Tg(-17.6isl2b:GFP)^zc7^*, *Tg(-17.6isl2b:mCherry-HsHRAS)^zc23^*, *Tg(-17.6isl2b:gap43-GFP)^zc20^*,	Labels all or the vast majority of RGCs	[99]
*Pou4f1: GFP *(*Brn3a:GFP*), *Pou4f1:Gal4 *(*Brn3a:Gal4*)	*Tg(pou4f1-hsp70l:gfp)^rw0110b^*	Labels RGCs. Also labels many PVNs, including glutamatergic PVPNs with ipsilateral axons to the hindbrain and GABAergic neurons with tectotectal axons	[27]
*Gal4s1013t*	*Et(-1.5hsp70l:gal4-vp16)s1013t*	Drives expression in all neurons and glia of the tectum	[20]
*Gal4s1038t*	*Et(fos:gal4-vp16)s1038t*	Drives expression in PVPNs of the posterior tectum	[20]
*Gal4s1156t*	*Et(-1.5hsp70l:gal4-vp16)s1156t*	Drives expression in very few tectal neurons, including most SINs	[54]
*Gal4s1101t*	*Et(e1b:gal4-vp16)s1101t*	Drives expression in almost all neurons of the CNS; 'pan-neural'	[55,59,60]

CNS, central nervous system.

Important contributions to the renaissance of interest in the tectum's inner workings have also been made in *Xenopus *tadpoles. We will be lumping efforts in fish and frog together here, as they are truly complementary, each capitalizing on specific experimental advantages of the two systems.

## Spatial patterning of information flow in the optic tectum

The zebrafish larval tectum is roughly divided into two regions, a deep cell body layer, the stratum periventriculare (SPV), and a superficial neuropil area, which contains the dendrites and axons of tectal neurons, a sparse assortment of tectal interneurons and afferent axons arriving at the tectum, chiefly from the retina (Figure 1b; colored circles indicate the diverse tectal cell types - see next section). Tectal processing begins with visual signals transmitted via the axons of RGCs. These axons enter the zebrafish tectal neuropil from the anterior end at six levels corresponding to the six retinorecipient laminae (Figure 1b) [10]. A similar pattern has been observed in adult teleosts [11]. As a strict rule, each RGC axon is targeted to a single lamina and arborizes exclusively in this lamina [12]. Most (80%) RGC axons innervate three sublayers of the stratum fibrosum et griseum superficiale (SFGS). A smaller number (15%) innervate the most superficial stratum opticum (SO). The remaining RGC axons (5%) project into the stratum griseum centrale (SGC) and into the interface between the stratum album centrale and the SPV (SAC/SPV). Each retinorecipient lamina is topographically organized: retinal axons project into the plane of each layer in a visuotopic order, such that the retinotectal map is in fact an array of six parallel maps stacked on top of each other. Objects in the forward visual field of the contralateral eye are represented in anterior tectum, whereas objects behind the fish are mapped to the posterior tectum. Objects in the upper visual field activate the dorsal (medial) tectum, whereas the ventral (lateral) tectum responds to visual stimuli from below the fish. This fine-grained map is thought to allow the localization of a stimulus in the visual field.

Several general rules govern information processing in the fish tectum. Information flows primarily from the superficial layers to the deeper layers. The vast majority of retinal afferents enter the superficial layers of the tectum, where they make excitatory (glutamatergic) synaptic connections with the dendrites of tectal interneurons. The information then travels along the vertically oriented dendrites of the periventricular neurons (PVNs) to the deeper layers [13]. As a demonstration of this, Kinoshita *et al. *[14] labeled tectal slices of adult rainbow trout with a voltage-sensitive dye and imaged the propagation of activity as current was applied to the anterior pole of the SO and SFGS. This cross-sectional view of the working tectum confirmed that the wave of depolarization proceeds in a stereotyped pattern. Fast depolarization travels anterior to posterior in the SO and SFGS, presumably along the paths of RGC axons. At each point along the anterior-posterior axis, a slower vertical depolarization is triggered, proceeding radially to the deeper SGC and SAC.

In the deeper neuropil layers, information is transmitted from the axons of interneurons to other interneurons or to tectal projection neurons that send axons to premotor areas in the midbrain and hindbrain. Intratectal connections are inhibitory (releasing the neurotransmitter γ-aminobutyric acid (GABA) and thus called GABAergic) or excitatory (releasing glutamate; glutamatergic). In addition, a small percentage of PVNs are cholinergic (releasing acetylcholine). Tectal outputs from the deeper neuropil layers are wired to the appropriate combination of premotor nuclei to govern behavioral responses. The cell bodies of most tectal neurons are spatially removed from the site of actual processing, which seems to take place exclusively in the neuropil. The cell body is not required as an intermediate between input and output because of the peculiar 'monopolar' morphology of fish tectal cells, which are reminiscent of insect neurons. The dendritic segments of the neurites are contiguous with the axonal segments. In the voltage-sensitive dye recordings mentioned above [14], the SPV was not detectably activated, suggesting that the bulk of activity 'fades' in the proximal neurites before it reaches the cell bodies.

This cellular architecture probably has functional implications. Bollmann *et al. *[15] imaged individually dye-loaded tectal neurons in *Xenopus *tadpoles. Their study demonstrated that visually evoked dendritic calcium elevations are unevenly elicited across individual dendritic trees in a pattern consistent with the retinotopic map. Given that many tectal neurons have axons that emerge from among the dendritic branches, different levels of activation across the dendritic arbor might influence neuronal output differently. If so, dendrites nearer the initial axon segment would have more influence than more distal branches in spike generation. It is not clear how this bias, favoring certain retinotopic positions over others, might contribute to the shape of the PVN's receptive field.

Studies of genetic mutants have helped to identify mechanisms that govern the processing of visual information in the zebrafish tectum (Table 2). One example is the *blumenkohl *mutant, which shows a selective deficit in the capture of small prey items (but not large ones). This impairment is due to a deletion of vesicular glutamate transporter 2, encoded by the *vglut2a *gene. In response to decreased levels of glutamate at retinotectal synapses, the arbors of retinal axons become enlarged, resulting in an increase of the receptive fields of tectal neurons [16]. Accurate processing of visual stimuli requires spatially precise vertical streams of activity that subsequently recruit small subpopulations of projection neurons to initiate a motor response. In *blumenkohl *mutants, these parallel processing streams are less precisely aligned owing to a greater overlap of receptive fields among neighboring tectal PVNs. This seems to degrade either visual acuity or motor control (or both).

## Cell type diversity and complexity of tectal responses

Early electrophysiological recordings found heterogeneous responses among tectal neurons in adult zebrafish [17]. Some neurons were responsive to looming stimuli, others to moving edges or to objects of a certain size range. The colored circles in the schematic drawing in Figure 1b represent this diversity. Calcium imaging studies refined this work, showing that these distinct tuning properties arise early and are largely constant during embryonic and early larval development [18]. For these studies, larvae had their tecta loaded with a calcium indicator dye and were mounted with a miniature liquid-crystal display (LCD) screen for projecting images to the eye, and calcium signals from tectal cells were recorded by two-photon laser-scanning microscopy. PVNs could be sorted into numerous types according to their tuning profiles. Although some were broadly responsive, showing spontaneous and sustained activity in the dark, others were altogether unresponsive to the visual stimuli tested. However, the majority of PVNs were sensitive to spots in the visual field, with optimal responses to either stationary flashing spots, moving spots regardless of their size or direction, or small spots moving in particular directions. Thus, PVN ensemble activity probably encodes information about the location, size and movement of small objects in the visual field, evidently supporting the behavioral functions of the tectum.

**Figure 2:**
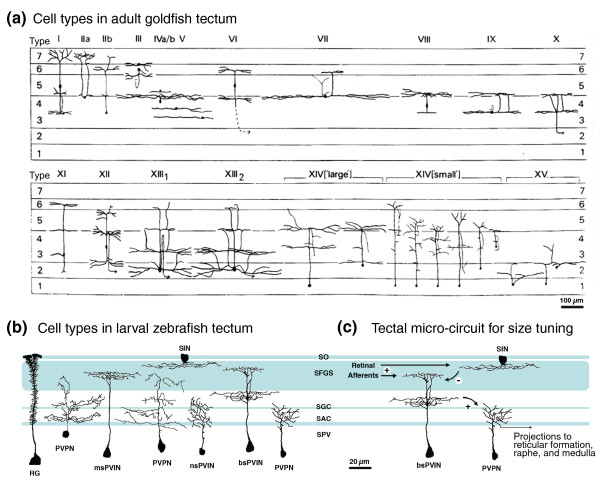
**Cell type diversity and (some) functional connectivity of the fish optic tectum**. **(a) **Cells described from classical Golgi studies in the adult goldfish tectum [19]. Fourteen types of neuron were identified on the basis of cell body position and morphology. Modified from [19]. **(b) **A sampling of neuron morphologies observed in the larval zebrafish tectum using 'genetic Golgi' methods. These include: radial glia (RG), periventricular projection neurons (PVPNs), periventricular interneurons (PVINs) and superficial interneurons (SINs). Retinorecipient laminae in the tectum are indicated by shading. Note the diverse dendrite morphologies of both projection neurons and interneurons in the tectum. In particular, PVINs have been observed containing arbors that are non-stratified (nsPVINs), mono-stratified (msPVINs) or bi-stratified (bsPVINs). **(c) **Hypothetical neural circuit responsible for size tuning of PVNs in the optic tectum [36]. Retinal afferents targeting the superficial layers of the SO and SFGS form excitatory synapses onto PVINs containing superficial dendrites and an axonal arbor in a deeper layer. These PVINs may mediate the vertical flow of excitation in response to small visual stimuli by activating PVPNs with dendrites located in deeper neuropil layers. In contrast, large visual stimuli additionally activate SIN cells, which inhibit the PVIN-mediated vertical flow of information to PVPNs.

A landmark neuro-anatomical study, using the Golgi labeling technique, in adult goldfish, a species closely related to zebrafish (both are in the family Cyprinidae), catalogued tectal neuron types on the basis of cell body location and neurite arborization pattern [19] (Figure 2a). (Golgi-labeling is a classical neuroanatomical technique for sparsely labeling neurons; it shares a name - from the physician scientist Camillo Golgi - with the cellular compartment, but is functionally unrelated.) Anatomical surveys of transgenically labeled neurons have now extended this classical work to larval zebrafish. In a screen, our group [20,21] identified three enhancer trap lines with strong and fairly specific expression of our *Gal4 *trap construct in tectal cells (Table 3). To examine individual neuron morphologies, we crossed these fish with carriers of a highly variegated *UAS:mGFP *construct contained within the *Brn3c:Gal4, UAS:mGFP *(*BGUG*) transgene (Table 3; GFP refers to green fluorescent protein and Brn3c to a member of the POU domain transcription factor family that is expressed in specific neurons). This method allows the visualization of single or sparse neurons with a membrane-targeted GFP. A distinct subset of these tectal neurons has been further characterized using a *Dlx4/5:GFP *transgenic reporter (ER, SJ Smith and HB, unpublished work). Together with single-cell electroporations labeling random subsets of tectal neurons with GFP [22], such 'genetic Golgi' stains have yielded a preliminary catalog of neuron types in larval zebrafish tectum (Figure 2b). Importantly, many of these neuron types resemble miniature versions of those described in the adult goldfish (compare Figure 2a and 2b) and other teleosts [23,24].

A quarter of the neurons in our survey [20,21] have cell bodies in the SPV, radially oriented dendrites that reach to the superficial, retinorecipient layers and a local axon. We call this group the periventricular interneurons (PVINs). Little is known about their function. A substantial fraction of PVINs are GABAergic (the rest being glutamatergic or cholinergic), and these may filter incoming signals by inhibiting responses to non-salient stimuli. Feedforward inhibitory connections need to be in place for gain control given the high ratio of retinotectal axons to tectal efferents (neurons projecting from the tectum), estimated to be between 30:1 and 100:1 [25]. Most other periventricular neurons (70%) have axons exiting the tectum, reaching the hindbrain reticular formation, the medulla or the Raphe nucleus [20]. As a rule, these periventricular projection neurons (PVPNs) have dendrite arbors in the deep and intermediate regions of the neuropil, but not in the superficial SO/SFGS zone [20,25,26]. This morphology reinforces the observation that information flows chiefly from superficial to deep [13] and further suggests that processed, rather than raw, visual information governs tectal output.

A study [27] of efferent projections from the deep layers of the tectal neuropil to the hindbrain suggests that spatially patterned tectal outputs may help coordinate motor responses. The tectobulbar tract is composed of ipsilateral and contralateral projections to premotor structures in the hindbrain reticular formation. Hindbrain target neurons, in turn, project to primary motor neurons in the spinal cord. Sato *et al. *[27] used a clever combination of Gal4/UAS and Cre/LoxP systems to label small numbers of PVPNs, allowing a direct comparison of retinotopic position of tectal cell bodies with the hindbrain targets of their axons. Each mini-region of the tectum projects axons to a wide array of hindbrain segments (rhombomeres); for example, one area the size of a single retinotectal arbor had projections to almost all rhombomeres. These observations support a model in which tectal output from a small region reaches multiple premotor sites in order to coordinate a full body response.

The topographic organization of tectofugal projections to the reticular formation is functionally important, as shown in mammals, amphibians and fish [28-32]. In goldfish, anterior tectal efferents preferentially innervate midbrain sites that generate small horizontal eye movements, and posterior efferents innervate sites associated with large saccades (fast reset movements). In larval zebrafish, there is a similar mapping of tectal efferents onto the reticular formation [27]. Posterior tectal neurons are more likely to project to rhombomere 2, whereas middle to anterior neurons are more likely to innervate rhombomere 6. This suggests that the behavioral responses that are controlled by the reticular motor map are tailored to the location of the visual stimulus (as they should be). Although we do not know the identity or function of neurons in rhombomere 2 that receive input primarily from the posterior tectum, we predict that they have a role in executing a behavioral response, perhaps a large horizontal saccade or a turning response, to stimuli behind the animal.

## Filtering of visual inputs by tectal micro-circuits

The role of local inhibition in the tectum for visual discrimination has been brought to light in two studies. In the first, Ramdya and Engert [33] surgically removed one tectum from a developing zebrafish embryo, which resulted in bilateral retinal innervation of the remaining tectum. This allowed them to characterize the binocular response properties of normally monocular tectal neurons. As in monocular tectum, binocular tectal neurons sometimes responded to motion in a direction-selective manner. Even a stripped-down motion stimulus, consisting of a dot jumping between two movie frames from left to right, generated a response. The authors exploited this unnatural binocular response to ask how motion sensitivity is generated in the tectum. They created an artificial 'motion stimulus' visible only to a binocular cell by parsing the dot's jump between the two eyes: one movie frame was shown only to the right eye and the other frame only to the left eye. Interestingly, this two-frame movie was sufficient to stimulate direction-sensitive tectal neurons. Given that neither retina's signal, on its own, could encode motion direction - each was shown a flashing, stationary dot - it can be concluded that circuitry intrinsic to the tectum underlies this sensitivity. These results are consistent with a model in which a direction-sensitive cell responsive to motion in the anterior direction is flanked anteriorly by retinorecipient cells that inhibit its activity. The direction-sensitive cell is therefore inhibited by its anterior neighbors and shows reduced activity when activated by a stimulus moving in a posterior direction across its receptive field, but responds more vigorously to an anteriorly moving spot. A similar model partially accounts for the directional selectivity seen in neurons of mammalian visual cortex [34,35]. The Ramdya and Engert study [33] showed that such a direction-sensitive circuit exists in the zebrafish tectum and that it is probably hardwired (genetically specified).

The role of a specific, anatomically identified class of tectal inhibitory interneurons has recently emerged in another calcium imaging study carried out in our laboratory [36]. This work used the enhancer trap lines generated by Scott *et al. *[21] to express the genetically encoded calcium indicator GCaMP in specific populations of tectal cells in order to image response dynamics in different tectal layers. As previously observed in the superior colliculus of mammals and corroborated in the larval zebrafish [18] (see above), many tectal neurons respond most strongly to small spots or bars in visual space. The new results identify a synaptic basis for this small-spot bias. When presented on a small LCD screen, all visual stimuli registered post-synaptic responses in the superficial neuropil (SO and SFGS), but only for spatially restricted stimuli were these signals fully propagated into the deeper neuropil (SGC and SAC). Tectal administration of bicuculline eliminated this size selectivity - in the presence of this GABA antagonist, large (50°) stimuli elicited responses in both superficial and deep neuropil. This implicates GABA-based inhibition in tectal filtering [36].

Further work was able to identify the cell type responsible for the selectivity [36]. With another Gal4 line, a type of interneuron called a superficial inhibitory neuron (SIN) could be labeled, whose cell body resides between SO and SFGS (Figure 2c). These cells have broad arbors in the SFGS, are GABAergic, and are probably homologous to Meek and Schellart's type III neurons (Figure 2a) [19]. Calcium imaging showed that these cells have the unusual property of responding to full-screen stimuli (and not to small stimuli). These data raised the possibility that SINs may be mediating the small spot selectivity of tectal filtering. To demonstrate their necessity in this process, SINs were photo-ablated with KillerRed [37]. This lesion had a similar effect to the application of bicuculline; deep tectal neuropil layers responded to large and small stimuli alike. Moreover, silencing of synaptic transmission by SINs with the tetanus toxin light chain impaired the fish's prey capture behavior, but not optomotor responses, a behavior independent of the tectum [38]. The new experiments [36] are the first elucidation of the role of a morphologically and genetically designated cell type in tectal processing.

## Remaining questions and emerging approaches

The tectum integrates and processes visual information for export to premotor targets. Several steps in this sensorimotor transformation are still mysterious. The rules governing the PVIN to PVPN transmission are unknown, as are the contributions of afferent inputs to the tectum from diverse brain regions and sensory modalities [39-41]. And although efferent targets have been identified anatomically, we know little of the spatial or temporal patterns of tectal output activity. Even more mysteriously, tectal circuitry shows oscillations of activity in response to a periodic visual stimulus, which can continue long (tens of seconds) after the stimulus has stopped. These entrained mental 'reverberations' can even drive rhythmic motor activity [42]. We do not know which neuronal networks carry these oscillations, and whether they could potentially provide a substrate for working memory. A complete catalog of cell types, together with a comprehensive description of their connections within the tectum and beyond, will be useful to deduce this and other computations carried out by tectal micro-circuits.

Given the tectum's superficial position in the dorsal brain and the transparency of larval zebrafish, these questions can now be addressed using *in vivo *imaging and emerging optogenetic tools (reviewed in [43]). A large number of genetically encoded fluorescent and luminescent indicators of calcium concentration [42,44-46], voltage [47-49] or neurotransmitter release [50-52] are available, some of which have already proven effective in zebrafish [53,54]. Activating proteins, such as channelrhodopsins and LiGluR, and silencing proteins, including halorhodopsin, have recently been used in zebrafish to link targeted neurons conclusively to their roles in simple behaviors [55-60]. To take full advantage of these methods, more specific lines expressing transgenes in subsets of tectal neurons will have to be generated. Extrapolating from the rapid pace of recent discoveries, we expect that many of the anatomical components of the tectal circuitry will soon be understood in terms of their function in visual perception and behavior.

The mammalian superior colliculus also receives topographically organized retinal inputs and, like the tectum, has a stratified architecture that is principally visual in the superficial layers and multimodal with motor outputs in deeper layers [61]. Although extrinsic collicular circuits, including a number of command projections from the forebrain, are better characterized in mammals and birds than in zebrafish, understanding of the micro-circuitry is sketchy. In this way, investigations in different vertebrate species are complementary, and findings from one enable targeted studies in the other. Mammalian equivalents to SINs would be an appealing first target. The means by which PVINs and other tectal interneurons filter visual information could also be shared between fish and mammals, and as these processes are elucidated in the tectum, they will probably provide insights into collicular function.

More broadly, studies in the tectum have provided glimpses of how a three-dimensional array of neurons, whose architecture is simple by central nervous system standards, can filter input, represent visual space and detect motion. Genetic, behavioral and optical access to the tectum should allow the underlying cellular mechanisms to be described in the coming years. As these details emerge, we will probably learn important fundamentals of how diverse neural networks function.

## References

[B1] Van Essen DC Anderson CH Felleman DJ Information processing in the primate visual system: an integrated systems perspective Science 1992 255 419 423 10.1126/science.1734518 1734518

[B2] Fite KV Pretectal and accessory-optic visual nuclei of fish, amphibia and reptiles: theme and variations Brain Behav Evol 1985 26 71 90 10.1159/000118769 3907745

[B3] Ewert JP Buxbaum-Conradi H Dreisvogt F Glagow M Merkel-Harff C Röttgen A Schörg-Pfeiffer E Schwippert WW Neural modulation of visuomotor functions underlying prey-catching behaviour in anurans: perception, attention, motor performance, learning Comp Biochem Physiol A Mol Integr Physiol 2001 128 417 461 10.1016/S1095-6433(00)00333-0 11246037

[B4] Baier H Zebrafish on the move: towards a behavior-genetic analysis of vertebrate vision Curr Opin Neurobiol 2000 10 451 455 10.1016/S0959-4388(00)00116-1 10981613

[B5] Friedrich RW Jacobson GA Zhu P Circuit neuroscience in zebrafish Curr Biol 2010 in press 10.1016/j.cub.2010.02.039 21749961

[B6] Gahtan E Baier H Of lasers, mutants, and see-through brains: functional neuroanatomy in zebrafish J Neurobiol 2004 59 147 161 10.1002/neu.20000 15007833

[B7] Portugues R Engert F The neural basis of visual behaviors in the larval zebrafish Curr Opin Neurobiol 2009 19 644 647 10.1016/j.conb.2009.10.007 19896836 PMC4524571

[B8] Burrill JD Easter SS Jr Development of the retinofugal projections in the embryonic and larval zebrafish (*Brachydanio rerio*) J Comp Neurol 1994 346 583 600 10.1002/cne.903460410 7983245

[B9] Ullén F Deliagina TG Orlovsky GN Grillner S Visual pathways for postural control and negative phototaxis in lamprey J Neurophysiol 1997 78 960 976 9307127 10.1152/jn.1997.78.2.960

[B10] Xiao T Roeser T Staub W Baier H A GFP-based genetic screen reveals mutations that disrupt the architecture of the zebrafish retinotectal projection Development 2005 132 2955 2967 10.1242/dev.01861 15930106

[B11] Vanegas H Ito H Morphological aspects of the teleostean visual system: a review Brain Res 1983 287 117 137 6315186 10.1016/0165-0173(83)90036-x

[B12] Xiao T Baier H Lamina-specific axonal projections in the zebrafish tectum require the type IV collagen Dragnet Nat Neurosci 2007 10 1529 1537 10.1038/nn2002 17982451

[B13] Kinoshita M Ito E Roles of periventricular neurons in retinotectal transmission in the optic tectum Prog Neurobiol 2006 79 112 121 10.1016/j.pneurobio.2006.06.002 16901616

[B14] Kinoshita M Ueda R Kojima S Sato K Watanabe M Urano A Ito E Multiple-site optical recording for characterization of functional synaptic organization of the optic tectum of rainbow trout Eur J Neurosci 2002 16 868 876 10.1046/j.1460-9568.2002.02160.x 12372023

[B15] Bollmann JH Engert F Subcellular topography of visually driven dendritic activity in the vertebrate visual system Neuron 2009 61 895 905 10.1016/j.neuron.2009.01.018 19323998 PMC2892759

[B16] Smear MC Tao HW Staub W Orger MB Gosse NJ Liu Y Takahashi K Poo Mm Baier H Vesicular glutamate transport at a central synapse limits the acuity of visual perception in zebrafish Neuron 2007 53 65 77 10.1016/j.neuron.2006.12.013 17196531 PMC1828615

[B17] Sajovic P Levinthal C Visual cells of zebrafish optic tectum: mapping with small spots Neuroscience 1982 7 2407 2426 10.1016/0306-4522(82)90204-4 7177381

[B18] Niell CM Smith SJ Functional imaging reveals rapid development of visual response properties in the zebrafish tectum Neuron 2005 45 941 951 10.1016/j.neuron.2005.01.047 15797554

[B19] Meek J Schellart NAM A golgi study of goldfish optic tectum J Comp Neurol 1978 182 89 111 10.1002/cne.901820107 81216

[B20] Scott EK Baier H The cellular architecture of the larval zebrafish tectum, as revealed by gal4 enhancer trap lines Front Neural Circuits 2009 3 13 10.3389/neuro.04.013.2009 19862330 PMC2763897

[B21] Scott EK Mason L Arrenberg AB Ziv L Gosse NJ Xiao T Chi NC Asakawa K Kawakami K Baier H Targeting neural circuitry in zebrafish using GAL4 enhancer trapping Nat Methods 2007 4 323 326 17369834 10.1038/nmeth1033

[B22] Gosse NJ Nevin LM Baier H Retinotopic order in the absence of axon competition Nature 2008 452 892 895 10.1038/nature06816 18368050 PMC2885002

[B23] Heiligenberg W Rose GJ The optic tectum of the gymnotiform electric fish, *Eigenmannia*: labeling of physiologically identified cells Neuroscience 1987 22 331 340 10.1016/0306-4522(87)90224-7 3627446

[B24] Kinoshita M Ito E Urano A Ito H Yamamoto N Periventricular efferent neurons in the optic tectum of rainbow trout J Comp Neurol 2006 499 546 564 10.1002/cne.21080 17029270

[B25] Meek J Functional anatomy of the tectum mesencephali of the goldfish. An explorative analysis of the functional implications of the laminar structural organization of the tectum Brain Res 1983 287 247 297 6362772 10.1016/0165-0173(83)90008-5

[B26] Meek J A Golgi-electron microscopic study of goldfish optic tectum. I. Description of afferents, cell types, and synapses J Comp Neurol 1981 199 149 173 10.1002/cne.901990202 7251937

[B27] Sato T Hamaoka T Aizawa H Hosoya T Okamoto H Genetic single-cell mosaic analysis implicates ephrinB2 reverse signaling in projections from the posterior tectum to the hindbrain in zebrafish J Neurosci 2007 27 5271 5279 10.1523/JNEUROSCI.0883-07.2007 17507550 PMC6672335

[B28] Cohen B Buttner-Ennever JA Projections from the superior colliculus to a region of the central mesencephalic reticular formation (cMRF) associated with horizontal saccadic eye movements Exp Brain Res 1984 57 167 176 10.1007/BF00231143 6519224

[B29] Herrero L Pérez P Núnez Abades P Hardy O Torres B Tectotectal connectivity in goldfish J Comp Neurol 1999 411 455 471 10.1002/(SICI)1096-9861(19990830)411:3<455::AID-CNE8>3.0.CO;2-7 10413779

[B30] Herrero L Rodríguez F Salas C Torres B Tail and eye movements evoked by electrical microstimulation of the optic tectum in goldfish Exp Brain Res 1998 120 291 305 10.1007/s002210050403 9628416

[B31] Pérez-Pérez MP Luque MA Herrero L Nuñez-Abades PA Torres B Connectivity of the goldfish optic tectum with the mesencephalic and rhombencephalic reticular formation Exp Brain Res 2003 151 123 135 10.1007/s00221-003-1432-6 12748838

[B32] Salas C Herrero L Rodriguez F Torres B Tectal codification of eye movements in goldfish studied by electrical microstimulation Neuroscience 1997 78 271 288 10.1016/S0306-4522(97)83048-5 9135107

[B33] Ramdya P Engert F Emergence of binocular functional properties in a monocular neural circuit Nat Neurosci 2008 11 1083 1090 10.1038/nn.2166 19160507 PMC2958220

[B34] Clifford CWG Ibbotson MR Fundamental mechanisms of visual motion detection: models, cells and functions Prog Neurobiol 2002 68 409 437 10.1016/S0301-0082(02)00154-5 12576294

[B35] Livingstone MS Mechanisms of direction selectivity in macaque V1 Neuron 1998 20 509 526 10.1016/S0896-6273(00)80991-5 9539125

[B36] Del Bene F Wyart C Robles E Tran A Looger L Scott EK Isacoff EY Baier H Filtering of visual information in the tectum by an identified neural circuit Science 2010 in press 10.1126/science.1192949 PMC3243732 21030657

[B37] Bulina ME Chudakov DM Britanova OV Yanushevich YG Staroverov DB Chepurnykh TV Merzlyak EM Shkrob MA Lukyanov S Lukyanov KA A genetically encoded photosensitizer Nat Biotechnol 2006 24 95 99 10.1038/nbt1175 16369538

[B38] Roeser T Baier H Visuomotor behaviors in larval zebrafish after GFP-guided laser ablation of the optic tectum J Neurosci 2003 23 3726 3734 12736343 10.1523/JNEUROSCI.23-09-03726.2003 PMC6742205

[B39] Deeg KE Sears IB Aizenman CD Development of multisensory convergence in the *Xenopus *optic tectum J Neurophysiol 2009 102 3392 3404 10.1152/jn.00632.2009 19793878 PMC2804420

[B40] Hiramoto M Cline HT Convergence of multisensory inputs in *Xenopus *tadpole tectum Dev Neurobiol 2009 69 959 971 10.1002/dneu.20754 19813244 PMC2902241

[B41] Pérez-Pérez MP Luque MA Herrero L Nuñez-Abades PA Torres B Afferent connectivity to different functional zones of the optic tectum in goldfish Vis Neurosci 2003 20 397 410 10.1017/S0952523803204053 14658768

[B42] Sumbre G Muto A Baier H Poo MM Entrained rhythmic activities of neuronal ensembles as perceptual memory of time interval Nature 2008 456 102 106 10.1038/nature07351 18923391 PMC2896960

[B43] Luo L Callaway EM Svoboda K Genetic dissection of neural circuits Neuron 2008 57 634 660 10.1016/j.neuron.2008.01.002 18341986 PMC2628815

[B44] Chi NC Shaw RM Jungblut B Huisken J Ferrer T Arnaout R Scott I Beis D Xiao T Baier H Jan LY Tristani-Firouzi M Stainier DY Genetic and physiologic dissection of the vertebrate cardiac conduction system PLoS Biol 2008 6 e109 10.1371/journal.pbio.0060109 18479184 PMC2430899

[B45] Li J Mack JA Souren M Yaksi E Higashijima SI Mione M Fetcho JR Friedrich RW Early development of functional spatial maps in the zebrafish olfactory bulb J Neurosci 2005 25 5784 5795 10.1523/JNEUROSCI.0922-05.2005 15958745 PMC6724871

[B46] Naumann EA Kampff AR Prober DA Schier AF Engert F Monitoring neural activity with bioluminescence during natural behavior Nat Neurosci 2010 13 513 520 10.1038/nn.2518 20305645 PMC2846983

[B47] Lundby A Mutoh H Dimitrov D Akemann W Knöpfel T Engineering of a genetically encodable fluorescent voltage sensor exploiting fast Ci-VSP voltage-sensing movements PLoS ONE 2008 3 e2514 10.1371/journal.pone.0002514 18575613 PMC2429971

[B48] Tsutsui H Higashijima S Miyawaki A Okamura Y Visualizing voltage dynamics in zebrafish heart J Physiol 2010 588 2017 2021 10.1113/jphysiol.2010.189126 20421282 PMC2911208

[B49] Tsutsui H Karasawa S Okamura Y Miyawaki A Improving membrane voltage measurements using FRET with new fluorescent proteins Nat Methods 2008 5 683 685 10.1038/nmeth.1235 18622396

[B50] Dreosti E Odermatt B Dorostkar MM Lagnado L A genetically encoded reporter of synaptic activity *in vivo* Nat Methods 2009 6 883 889 10.1038/nmeth.1399 19898484 PMC2859341

[B51] Ng M Roorda RD Lima SQ Zemelman BV Morcillo P Miesenböck G Transmission of olfactory information between three populations of neurons in the antennal lobe of the fly Neuron 2002 36 463 474 10.1016/S0896-6273(02)00975-3 12408848

[B52] Yuste R Miller RB Holthoff K Zhang S Miesenbock G Synapto-pHluorins: chimeras between pH-sensitive mutants of green fluorescent protein and synaptic vesicle membrane proteins as reporters of neurotransmitter release Methods Enzymol 2000 327 522 546 10.1016/S0076-6879(00)27300-X 11045007

[B53] Baier H Scott EK Genetic and optical targeting of neural circuits and behavior--zebrafish in the spotlight Curr Opin Neurobiol 2009 19 553 560 10.1016/j.conb.2009.08.001 19781935 PMC2787859

[B54] Scott EK The Gal4/UAS toolbox in zebrafish: new approaches for defining behavioral circuits J Neurochem 2009 110 441 456 10.1111/j.1471-4159.2009.06161.x 19457087

[B55] Szobota S Gorostiza P Del Bene F Wyart C Fortin DL Kolstad KD Tulyathan O Volgraf M Numano R Aaron HL Scott EK Kramer RH Flannery J Baier H Trauner D Isacoff EY Remote control of neuronal activity with a light-gated glutamate receptor Neuron 2007 54 535 545 10.1016/j.neuron.2007.05.010 17521567

[B56] Wyart C Bene FD Warp E Scott EK Trauner D Baier H Isacoff EY Optogenetic dissection of a behavioural module in the vertebrate spinal cord Nature 2009 461 407 410 10.1038/nature08323 19759620 PMC2770190

[B57] Douglass AD Kraves S Deisseroth K Schier AF Engert F Escape behavior elicited by single, channelrhodopsin-2-evoked spikes in zebrafish somatosensory neurons Curr Biol 2008 18 1133 1137 10.1016/j.cub.2008.06.077 18682213 PMC2891506

[B58] Zhu P Narita Y Bundschuh ST Fajardo O Scharer YP Chattopadhyaya B Bouldoires EA Stepien AE Deisseroth K Arber S Sprengel R Rijli FM Friedrich RW Optogenetic dissection of neuronal circuits in zebrafish using viral gene transfer and the Tet system Front Neural Circuits 2009 3 21 10.3389/neuro.04.021.2009 20126518 PMC2805431

[B59] Arrenberg AB Del Bene F Baier H Optical control of zebrafish behavior with halorhodopsin Proc Natl Acad Sci USA 2009 106 17968 17973 10.1073/pnas.0906252106 19805086 PMC2764931

[B60] Schoonheim PJ Arrenberg AB Del Bene F Baier H Optogenetic localization and genetic perturbation of saccade-generating neurons in zebrafish J Neurosci 2010 30 7111 7120 10.1523/JNEUROSCI.5193-09.2010 20484654 PMC3842466

[B61] May PJ The mammalian superior colliculus: laminar structure and connections Prog Brain Res 2006 151 321 378 full_text 16221594 10.1016/S0079-6123(05)51011-2

[B62] Easter SS Jr Nicola GN The development of vision in the zebrafish (*Danio rerio*) Dev Biol 1996 180 646 663 10.1006/dbio.1996.0335 8954734

[B63] Emran F Rihel J Dowling JE A behavioral assay to measure responsiveness of zebrafish to changes in light intensities J Vis Exp 2008 20 923 19078942 10.3791/923 PMC2879884

[B64] Kimmel CB Patterson J Kimmel RO The development and behavioral characteristics of the startle response in the zebra fish Dev Psychobiol 1974 7 47 60 10.1002/dev.420070109 4812270

[B65] Kokel D Bryan J Laggner C White R Cheung CY Mateus R Healey D Kim S Werdich AA Haggarty SJ Macrae CA Shoichet B Peterson RT Rapid behavior-based identification of neuroactive small molecules in the zebrafish Nat Chem Biol 2010 6 231 237 10.1038/nchembio.307 20081854 PMC2834185

[B66] Kay JN Finger-Baier KC Roeser T Staub W Baier H Retinal ganglion cell genesis requires lakritz, a Zebrafish atonal Homolog Neuron 2001 30 725 736 10.1016/S0896-6273(01)00312-9 11430806

[B67] Neuhauss SCF Biehlmaier O Seeliger MW Das T Kohler K Harris WA Baier H Genetic disorders of vision revealed by a behavioral screen of 400 essential loci in zebrafish J Neurosci 1999 19 8603 8615 10493760 10.1523/JNEUROSCI.19-19-08603.1999 PMC6783047

[B68] Emran F Rihel J Adolph AR Dowling JE Zebrafish larvae lose vision at night Proc Natl Acad Sci USA 2010 107 6034 6039 10.1073/pnas.0914718107 20224035 PMC2851871

[B69] Li L Dowling JE Zebrafish visual sensitivity is regulated by a circadian clock Vis Neurosci 1998 15 851 857 9764527 10.1017/s0952523898155050

[B70] Brockerhoff SE Hurley JB Janssen-Bienhold U Neuhauss SCF Driever W Dowling JE A behavioral screen for isolating zebrafish mutants with visual system defects Proc Natl Acad Sci USA 1995 92 10545 10549 10.1073/pnas.92.23.10545 7479837 PMC40648

[B71] Burgess HA Schoch H Granato M Distinct retinal pathways drive spatial orientation behaviors in zebrafish navigation Curr Biol 2010 20 381 386 10.1016/j.cub.2010.01.022 20153194 PMC3412192

[B72] Orger MB Baier H Channeling of red and green cone inputs to the zebrafish optomotor response Vis Neurosci 2005 22 275 281 10.1017/S0952523805223039 16079003

[B73] Maximino C Marques de Brito T Dias CA Gouveia A Jr Morato S Scototaxis as anxiety-like behavior in fish Nat Protoc 2010 5 209 216 10.1038/nprot.2009.225 20134420

[B74] Nicolson T Rusch A Friedrich RW Granato M Ruppersberg JP Nusslein-Volhard C Genetic analysis of vertebrate sensory hair cell mechanosensation: the zebrafish circler mutants Neuron 1998 20 271 283 10.1016/S0896-6273(00)80455-9 9491988

[B75] Beck JC Gilland E Tank DW Baker R Quantifying the ontogeny of optokinetic and vestibuloocular behaviors in zebrafish, medaka, and goldfish J Neurophysiol 2004 92 3546 3561 10.1152/jn.00311.2004 15269231

[B76] Rinner O Rick JM Neuhauss SCF Contrast sensitivity, spatial and temporal tuning of the larval zebrafish optokinetic response Invest Ophthalmol Vis Sci 2005 46 137 142 10.1167/iovs.04-0682 15623766

[B77] Bilotta J Effects of abnormal lighting on the development of zebrafish visual behavior Behav Brain Res 2000 116 81 87 10.1016/S0166-4328(00)00264-3 11090887

[B78] Orger MB Kampff AR Severi KE Bollmann JH Engert F Control of visually guided behavior by distinct populations of spinal projection neurons Nat Neurosci 2008 11 327 333 10.1038/nn2048 18264094 PMC2894808

[B79] Orger MB Smear MC Anstis SM Baier H Perception of Fourier and non-Fourier motion by larval zebrafish Nat Neurosci 2000 3 1128 1133 10.1038/80649 11036270

[B80] Dill LM The escape response of the zebra danio (*Brachydanio rerio*). I. The stimulus for escape Anim Behav 1974 22 711 722 10.1016/S0003-3472(74)80022-9

[B81] Li L Dowling JE A dominant form of inherited retinal degeneration caused by a non-photoreceptor cell-specific mutation Proc Natl Acad Sci USA 1997 94 11645 11650 10.1073/pnas.94.21.11645 9326664 PMC23565

[B82] Borla MA Palecek B Budick S O'Malley DM Prey capture by larval zebrafish: evidence for fine axial motor control Brain Behav Evol 2002 60 207 229 10.1159/000066699 12457080

[B83] Budick SA O'Malley DM Locomotor repertoire of the larval zebrafish: swimming, turning and prey capture J Exp Biol 2000 203 2565 2579 10934000 10.1242/jeb.203.17.2565

[B84] Gahtan E Tanger P Baier H Visual prey capture in larval zebrafish is controlled by identified reticulospinal neurons downstream of the tectum J Neurosci 2005 25 9294 9303 10.1523/JNEUROSCI.2678-05.2005 16207889 PMC6725764

[B85] Bass SLS Gerlai R Zebrafish (*Danio rerio*) responds differentially to stimulus fish: the effects of sympatric and allopatric predators and harmless fish Behav Brain Res 2008 186 107 117 10.1016/j.bbr.2007.07.037 17854920

[B86] Gerlai R Fernandes Y Pereira T Zebrafish (*Danio rerio*) responds to the animated image of a predator: towards the development of an automated aversive task Behav Brain Res 2009 201 318 324 10.1016/j.bbr.2009.03.003 19428651 PMC2885451

[B87] Engeszer RE Ryan MJ Parichy DM Learned social preference in zebrafish Curr Biol 2004 14 881 884 10.1016/j.cub.2004.04.042 15186744

[B88] Engeszer RE Wang G Ryan MJ Parichy DM Sex-specific perceptual spaces for a vertebrate basal social aggregative behavior Proc Natl Acad Sci USA 2008 105 929 933 10.1073/pnas.0708778105 18199839 PMC2242707

[B89] McCann LI Koehn DJ Kline NJ The effects of body size and body markings on nonpolarized schooling behavior of zebra fish (*Brachydanio rerio*) J Psychol 1971 79 71 75 10.1080/00223980.1971.9923769 5116724

[B90] Miller N Gerlai R Quantification of shoaling behaviour in zebrafish (*Danio rerio*) Behav Brain Res 2007 184 157 166 10.1016/j.bbr.2007.07.007 17707522

[B91] Saverino C Gerlai R The social zebrafish: behavioral responses to conspecific, heterospecific, and computer animated fish Behav Brain Res 2008 191 77 87 10.1016/j.bbr.2008.03.013 18423643 PMC2486438

[B92] Turnell ER Mann KD Rosenthal GG Gerlach G Mate choice in zebrafish (*Danio rerio*) analyzed with video-stimulus techniques Biol Bull 2003 205 225 226 10.2307/1543265 14583542

[B93] Huang YY Tschopp M Neuhauss SCF Illusionary self-motion perception in zebrafish PLoS ONE 2009 4 e6550 10.1371/journal.pone.0006550 19672291 PMC2717804

[B94] Rick JM Horschke I Neuhauss SCF Optokinetic behavior is reversed in achiasmatic mutant zebrafish larvae Curr Biol 2000 10 595 598 10.1016/S0960-9822(00)00495-4 10837226

[B95] Nevin LM Taylor MR Baier H Hardwiring of fine synaptic layers in the zebrafish visual pathway Neural Dev 2008 3 36 10.1186/1749-8104-3-36 19087349 PMC2647910

[B96] Kay JN Link BA Baier H Staggered cell-intrinsic timing of ath5 expression underlies the wave of ganglion cell neurogenesis in the zebrafish retina Development 2005 132 2573 2585 10.1242/dev.01831 15857917

[B97] Masai I Lele Z Yamaguchi M Komori A Nakata A Nishiwaki Y Wada H Tanaka H Nojima Y Hammerschmidt M Wilson SW Okamoto H N-cadherin mediates retinal lamination, maintenance of forebrain compartments and patterning of retinal neurites Development 2003 130 2479 2494 10.1242/dev.00465 12702661

[B98] Zolessi FR Poggi L Wilkinson CJ Chien CB Harris WA Polarization and orientation of retinal ganglion cells *in vivo* Neural Dev 2006 1 2 10.1186/1749-8104-1-2 17147778 PMC1636330

[B99] Pittman AJ Law MY Chien CB Pathfinding in a large vertebrate axon tract: isotypic interactions guide retinotectal axons at multiple choice points Development 2008 135 2865 2871 10.1242/dev.025049 18653554 PMC2562560

[B100] Wagner HJ Wagner E Amacrine cells in the retina of a teleost fish, the roach (*Rutilus rutilus*): a Golgi study on differentiation and layering Philos Trans R Soc Lond B Biol Sci 1988 321 263 324 10.1098/rstb.1988.0094 2906747

